# High Refractive-Index Hybrids Consisting of Water-Soluble Matrices with Bipyridine-Modified Polyhedral Oligomeric Silsesquioxane and Lanthanoid Cations

**DOI:** 10.3390/polym12071560

**Published:** 2020-07-14

**Authors:** Kazunari Ueda, Takahiro Kakuta, Kazuo Tanaka, Yoshiki Chujo

**Affiliations:** 1 Department of Polymer Chemistry, Graduate School of Engineering, Kyoto University Katsura, Nishikyo-ku, Kyoto 615-8510, Japan; k321-mtmtys@nifty.com (K.U.); kakuta@se.kanazawa-u.ac.jp (T.K.); chujo@poly.synchem.kyoto-u.ac.jp (Y.C.); 2 Matsumoto Yushi-Seiyaku Co., Ltd., 2-1-3, Shibukawa-cho, Yao-City, Osaka 581-0075, Japan

**Keywords:** silsesquioxane, refractive index, Abbe number, lanthanoid, hybrid

## Abstract

We report high refractive-index (RI) films composed of polyhedral oligomeric silsesquioxane (SSQ) matrices and various lanthanoid cations. The SSQ matrices were constructed from octaammonium SSQ by connecting with bipyridine dicarboxylic acid, which is expected to capture cations. By modulating the feed ratio between SSQ and dicarboxylic acid, the series of the SSQ matrices were obtained with variable cross-linking ratios among the SSQ units. Thin transparent films were able to be prepared through the drop-casting method with the aqueous mixtures containing SSQ matrices and various kinds of lanthanoid salts up to 40 wt %. From RI measurements, it was revealed that the increase of the amount of the metal ion can significantly lift up the RI values. In particular, critical losses of Abbe numbers, which theoretically have the trade-off relationship toward increases in RI values, were hardly detected. This effect could be obtained by cation assembly in local spots that are assisted by SSQ.

## 1. Introduction

One of modern strategies for obtaining high refractive-index (RI) plastics is to prepare hybrid materials with polymer matrices and metal particles or ions [1]. Because it is easy to modulate properties in these hybrids by regulating types and concentrations of the loaded metal species, a wide variety of hybrid-based high RI materials have been developed [2,3,4,5,6]. In this purpose, lanthanoid ions, especially lanthanum, are also regarded as promising metal species for obtaining a high RI material because of the large effect to increase RIs [7]. However, it is strongly required to find comparable or substitute materials due to a drain on rare metal resources. In lanthanoids, cerium is known to be relatively abundant compared to other rare earth eleme1nts, while other types of elements have been desired for fabricating optical, electrical, and magnetic materials [8,9]. Thus, if we can establish to use cerium as a substituent of lanthanum, this technology should be helpful for solving the unbalance between supply and demand.

Polyhedral oligomeric silsesquioxane (SSQ) has attracted attention as a platform for preparing advanced optical materials [10,11,12,13,14,15,16]. By employing SSQ, the functional units can be accumulated in the compact space at the molecular level [17,18,19]. As a result, unique functions originating from the molecular assembly around SSQ can be observed. For example, by introducing SSQ into various media, thermal stability can be readily improved with soft materials that are not suitable for the hybridization with inorganic components [20,21,22]]. Moreover, Kudo et al. reported that the RI values of the polymer films can be lifted up by simply connecting the polymer chain ends with the rigid building blocks [23,24,25,26,]. Based on this finding, we also prepared the SSQ networks composed of oligosulfide linkers and detected the increase in the RI of conventional polymer films by blending these sulfur-containing SSQ networks without enlarging wavelength dependency of light refraction represented by an Abbe number [27]. More recently, similar increasing effects on RI without losses of Abbe numbers were also observed with the hybrids containing halogenated SSQ [28]. The transparent material having smaller wavelength dependency represented as a larger Abbe number is suitable in order to fabricate practical plastic lens. It should be mentioned that there is the trade-off relationship between high RI and Abbe number in theoretical. Meanwhile, only the increase in RI values can be induced by blending the sulfur-containing SSQ networks and halogenated SSQ, which suggests that the enhancement of local density around SSQ should affect only RIs.

We have recently proposed the facile preparation of functional hybrids by utilizing SSQ without troublesome sol-gel methods [29]. Simply by mixing designed SSQ to show good compatibility with polymer matrices, homogeneous materials with enhanced thermal stability, similarly to conventional hybrids, were obtained with conventional polymers [30,31,32,33,34,35]. In particular, the applicability of hybridization was able to be expanded to hydrophobic polymers, such as conjugated polymers, which are usually insoluble under general conditions in the sol‒gel reactions [36,37]. Thus, we regard SSQ as an element-block, which is a minimum functional unit that consists of heteroatom [38,39] for producing designable hybrids. Meanwhile, based on this hybridization strategy with SSQ, it is difficult to add another inorganic species due to high hydrophobicity of SSQ.

We designed the SSQ material having metal capturing units in order to obtain multi-component SSQ-based hybrids. In this paper, we report synthesis and properties of the water-soluble SSQ matrices that can retain various types of metal cations. Transparent films were obtained through the drop-casting with aqueous solutions of the matrices. Particularly, it was shown that large amounts of lanthanoid cations until 40 wt % can be loaded onto the film without losses of transparency. Furthermore, we found that the RIs of the films can be tuned by modulating the amount of cations without significant changes in the Abbe numbers. These results mean that the optical properties in the trade-off relationship are balanced in the SSQ matrices.

## 2. Results and Discussion

**Scheme 1:**
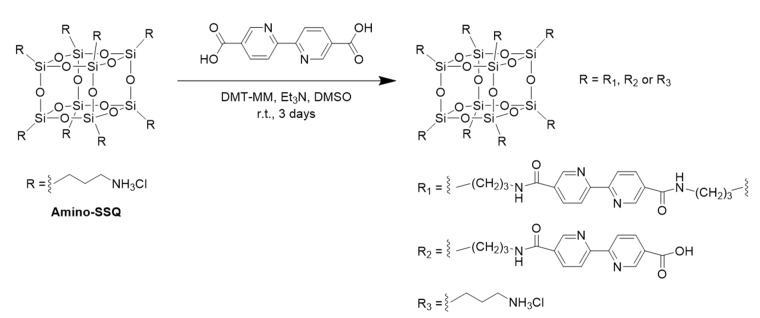
Synthesis of the silsesquioxane (SSQ) matrices.

Scheme 1 shows the design and preparation of the materials. In general, the introduction of metal cations to polymers is promised for effectively increasing the RIs of materials [7]. However, it is necessary to inhibit aggregation for maintaining transparency of materials. Therefore, we designed the SSQ matrices with the bipyridine linker to preserve dispersion states of metal cations homogeneously for obtaining good transparency as well as high RIs [40,41]. Furthermore, molecular assembly is easily accomplished by adsorption to SSQ [42,43,44,45,46,47,48,49]. It was also shown that lanthanoid cations can be efficiently captured in the presence of the appropriate ligand structures [50]. By applying the assembling effect of SSQ, it can be expected that an additional enhancement to RI values is expected without losses of Abbe numbers [27,28]. As a metal cation for loading onto the SSQ matrices, we selected the series of lanthanoid ions, which have been used as an additive for increasing RIs of polymer films. By changing the type of lanthanoid cations, we instead explored materials of lanthanum cation.

**Figure 1:**
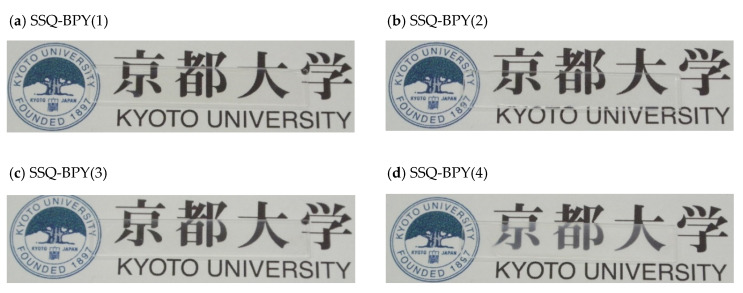
Appearances of the SSQ-BPY(*n*) films.

The series of the SSQ-containing films with various amounts of bipyridine linkers were prepared by modulating the feed ratios of bipyridine dicarboxylic acid to Amino-SSQ (Table 1) [51,52]. In the abbreviation, *n* denotes the mol% of SSQ-BPY(*n*) toward Amino-SSQ in the feed ratio. Owing to the good solubility of the products in water, we can estimate the cross-linking ratios between the SSQ unit and the bipyridine linker with 1H NMR spectroscopy [51,52]. After preparing film samples, it was shown that the SSQ-BPY(1) and SSQ-BPY(2) had good transparency, while slightly and quite white opaque films were obtained from SSQ-BPY(3) and SSQ-BPY(4), respectively (Figure 1). It is likely that the products with high bipyridine concentrations could involve large clusters in the films. Therefore, white turbidity could be generated. We also attempted to prepare several control materials, such as the mixture with Amino-SSQ and dicarboxylic acid without amide bonds and SSQ matrices, in the absence of bipyridine moieties. In both cases, by adding cations, critical phase separation occurred and homogeneous films that were suitable for optical measurements were no longer obtained. Moreover, although we were not able to determine the exact cross-linking rates between the SSQ units in ^1^H NMR spectra, good film-formability of the products proposes that the connection among the SSQ units could proceed. For the following experiments, we used transparent SSQ-BPY(2) film because of good transparency and film-formability.

**Figure 2:**
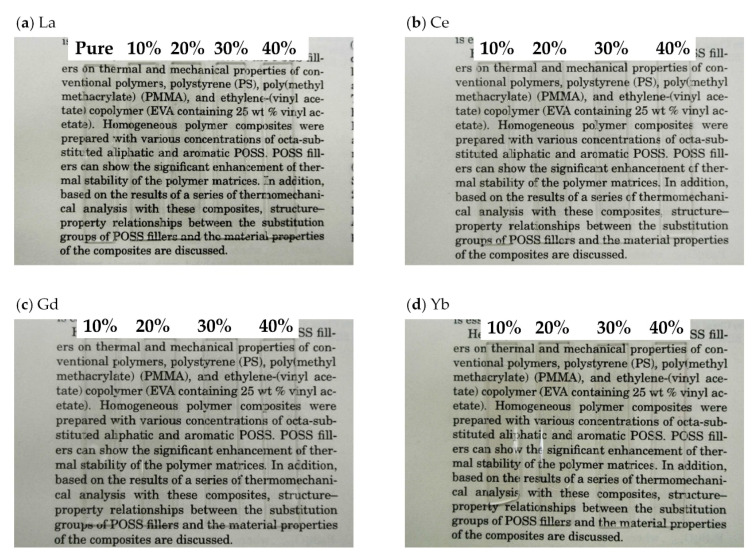
Appearances of the SSQ-BPY(2) films with 10–40 wt % lanthanoid salts.

**Figure 3:**
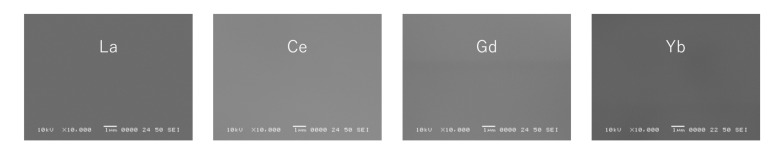
Scanning electron microscopy (SEM) images of SSQ-BPY(2) films containing various types of lanthanoid chloride salts (40 wt %). Scale bars represent 1 μm.

The SSQ-BPY(2) films containing 10‒40 wt % lanthanoid metal salts (La, Ce, Gd, Yb) also had good transparency, which was calculated as an averaged value of transmittance from 400 nm to 700 nm (Figure 2). According to the data, extremely high values (99%) were observed, even in the presence of 40 wt % cations (Figure S1). The surface morphologies of the films were investigated using scanning electron microscopy (SEM). Significant phase separation or aggregations were hardly observed in the SSQ-BPY(2) films, even with 40 wt % lanthanoid cations at the submicron scale (Figure 3). From these data, we concluded that SSQ films can have sufficient good homogeneous states for an evaluation of optical properties.

**Figure 4:**
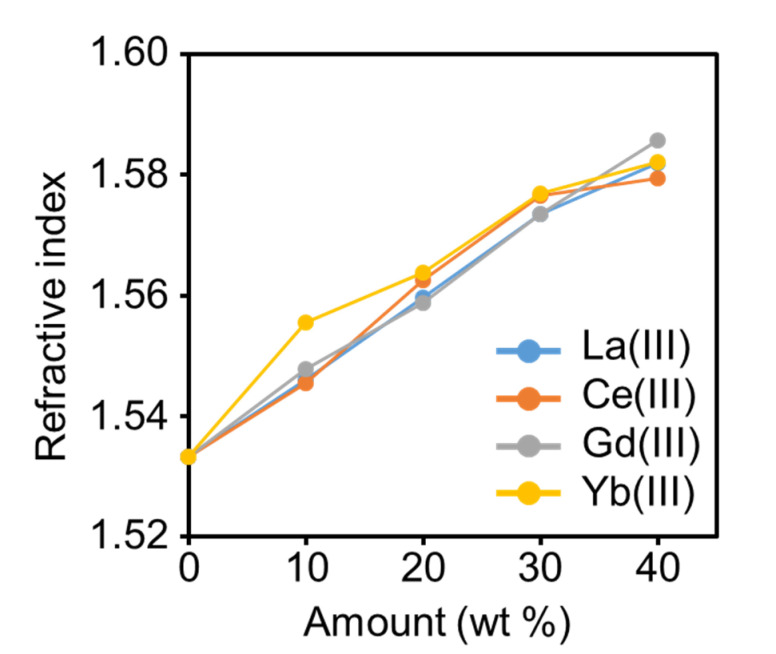
Refractive indices (*n*) of the SSQ-BPY(2) films containing various concentrations of lanthanoid chloride salts.

The RIs of the hybrid films with various concentrations of lanthanoid metal cations were measured with reflectance spectroscopy (Figure 4). The averaged values were calculated from the data set at five distinct points in the films (Table 2). The RIs of films in the presence of lanthanoid cations were higher than that of the pure SSQ-BPY(2) film. Further, similar values were obtained as compared to RIs of the films containing 40 wt % cations. In particular, the cerium-loaded films showed a similar degree of RIs with the films containing lanthanum cations, which indicated that cerium is instead a component of lanthanum for high RI materials in the SSQ matrix.

An Abbe number (*ν*_D_) was calculated from the wavelength dependency of RI according to Equation 1 (Table 2) [27,28]. Interestingly, less significant changes were observed, even by increasing the amounts of the lanthanoid cations in SSQ-BPY(2) matrices. The molecular assembly of cations around SSQ could play a major role in increasing the RIs of the films. Thus, all kinds of cation might show similar RIs with each concentration without critical losses in Abbe numbers.

Thermal stability was evaluated by measuring decomposition temperatures with 5 wt % weight losses (Figures S2 and S3). By adding 10 and 20 wt % cations, critical decreases in thermal stability were induced by loading lanthanum cations (−45 °C~−17 °C), while smaller decreasing effects were observed from the films with 40 wt % cerium cation (−12 °C) (Tables S1 and S2). It is implied that a smaller size of Ce^3+^ might be favorable for tight binding, followed by the suppression of thermal motions of matrices. These results positively support that cerium cation in SSQ matrices can instead be the element of lanthanum that is the typical component for fabricating high RI materials.

## 3. Conclusions

Here we describe that water-soluble SSQ matrices can work as a platform for fabricating high RI hybrid films. The transparent films can be obtained simply through casting methods with aqueous dispersions containing SSQ matrices and cations. Even with 40 wt % of lanthanoid cations, the SSQ films are able to maintain transparency as well as good homogeneity. The resulting films containing cations show high RI values that originate from lanthanoid cations. In particular, Abbe numbers of the hybrid films were maintained even with higher concentrations of cations. Another significant point is the fact that cerium can instead be a material for lanthanum in the SSQ matrix. When considering the difference in current production ratios between cerium and lanthanum, our strategy that is based on SSQ hybrid materials presented here could contribute to solving the problem on resource constraints of rare metals.

## Figures and Tables

**Table 1:** Optical properties of SSQ-BPY(2) containing lanthanoid salts.

Polymer	Feed Ratio of Dicarboxylic Acid (eq.)	Yield (%)	Crosslinking Ratio (%) *^a^*
SSQ-BPY(1)	1.0	82	13
SSQ-BPY(2)	2.0	75	24
SSQ-BPY(3)	3.0	72	35
SSQ-BPY(4)	4.0	63	42

*^a^* Determined from the ^1^H NMR spectra.

**Table 2:** Optical properties of SSQ-BPY(2) films containing lanthanoid salts.

Lanthanoid	wt %	*n*_D_	∆*n*_D_	*ν*_D_
none	-	1.5332		37
La	10	1.5461	0.0129	35
	20	1.5596	0.0264	37
	30	1.5735	0.0402	44
	40	1.5820	0.0488	39
Ce	10	1.5455	0.0123	35
	20	1.5625	0.0293	40
	30	1.5765	0.0433	41
	40	1.5794	0.0462	38
Gd	10	1.5478	0.0146	38
	20	1.5587	0.0255	39
	30	1.5734	0.0402	39
	40	1.5857	0.0525	35
Yb	10	1.5555	0.0223	39
	20	1.5638	0.0306	34
	30	1.5768	0.0436	39
	40	1.5820	0.0488	38

## Supplementary Materials

The following are available online at https://www.mdpi.com/2073-4360/12/7/1560/s1, Experimental Section, Figure S1: Transparency of the films containing 40 wt % (a) La(III), (b) Ce(III), (c) Gd(III) and (d) Yb(III) salts. Averaged values were calculated from 400 to 800 nm, Figure S2: TGA profiles of SSQ networks, Figure S3: TGA profiles of SSQ networks BPY(2) with various concentrations of metal cations, Table S1: Thermal stability of network polymers, Table S2: Thermal stability of hybrid films with variable cation concentrations.
