# Supplementary material for: High Refractive-Index Hybrids Consisting of Water-Soluble Matrices with Bipyridine-Modified Polyhedral Oligomeric Silsesquioxane and Lanthanoid Cations
Source: Polymers (Basel). 2020 Jul 14;12(7):1560. doi: 10.3390/polym12071560 (PMC7407817; doi:10.3390/polym12071560)
Supplement: Supplementary file 1 [file polymers-12-01560-s001.pdf]

Supporting Information

High Refractive-Index Hybrids Consisting of Water-Soluble Matrices with Bipyridine-Modified Polyhedral Oligomeric Silsesquioxane and Lanthanoid Cations

Kazunari Ueda[1,2], Takahiro Kakuta[1], Kazuo Tanaka[1]* and Yoshiki Chujo[1]

[1] Department of Polymer Chemistry, Graduate School of Engineering, Kyoto University Katsura, Nishikyo-ku, Kyoto 615-8510, Japan; k321-mtmtys@nifty.com (K.U.); kakuta@se.kanazawa-u.ac.jp (T.K.); chujo@poly.synchem.kyoto-u.ac.jp (Y.C.)
[2] Matsumoto Yushi-Seiyaku Co., Ltd., 2-1-3, Shibukawa-cho, Yao-City, Osaka 581-0075, Japan
* Correspondence: tanaka@poly.synchem.kyoto-u.ac.jp; Tel.: +81-75-383-2604; Fax: +81-75-383-2605

E-mail: tanaka@poly.synchem.kyoto-u.ac.jp

Experimental Section

General. NMR spectra were measured with a JEOL EX–400 (400 MHz for ^{1}H, 100 MHz for ^{13}C, and 80 MHz for ^{29}Si) spectrometer. Coupling constants (*J* value) are reported in Hertz. MASS spectra were obtained on a Thermo Fisher EXACTIVE for electron spray ionization (ESI). Scanning electron microscopy (SEM) images were performed using a JEOL JSM–5600 operated at an accelerating voltage of 10 kV. RIs (n_D) were determined with a reflectance spectroscopy film thickness meter (FE-3000, Otsuka Electronics Co., Ltd, Osaka, Japan) at 25 °C. The RIs of each wavelength in the visible region were calculated from the fitting of the data to the n-Cauchy approximation equation by entering the film thickness. The n_D values were obtained as the refractive index at 589.3 nm. Abbe numbers (ν_D) were calculated from the RIs at 468.1 (n_F), 656.3 (n_C), and 589.3 nm according to equation 1.

$$\nu_D = \frac{n_D - 1}{n_F - n_C} \quad (1)$$

Materials. Octa-substituted ammonium SSQ (Amino-SSQ)[7] and 2,2'-bipyridine-5,5'-dicarboxylic acid[8] were prepared according to the previous reports. 4-(4,6-dimethoxy-1,3,5-triazin-2-yl)-4-methylmorpholinium chloride (DMT-MM) (Wako Pure Chemical Industries, Ltd.), triethylamine (Et_3N) (Wako Pure Chemical Industries, Ltd.), hydrochloric acid (Wako Pure Chemical Industries, Ltd.), dimethyl sulfoxide (Wako Pure Chemical Industries, Ltd.), acetonitrile (Wako Pure Chemical Industries, Ltd.), gadolinium chloride hexahydrate (Wako Pure Chemical Industries, Ltd.), ytterbium chloride hexahydrate (Wako Pure Chemical Industries, Ltd.), lanthanum chloride heptahydrate (Nacalai Tesque), cerium chloride (Nacalai Tesque) were purchased and used for the assays without further purification.

Preparation of SSQ network. A typical procedure is shown here: 2,2'-bipyridine-5,5'-dicarboxylic acid (83 mg, 0.34 mmol) was dissolved in 15 mL DMSO. DMT-MM (208 mg, 0.75 mmol) was added to the solution, and the mixture was stirred at room temperature for 2 h. Triethylamine (96 μL, 0.69 mmol) and amino-SSQ (200 mg, 0.17 mmol) was added slowly to the mixture, and the solution was stirred at room temperature for 3 days. After stirring, the solution of the mixture was dropped to 50 mL of acetonitrile contained 0.1 wt% HCl. The precipitation was collected by filtration and washed with acetonitrile three times. The strained powder was dissolved in 20 mL of deionized water, and then the aqueous solution was filtrated to remove insoluble products. The aqueous solution was evaporated and dried *in vacuo* to give the SSQ network polymer cross-linked with bipyridine dicarboxylic acid. From the ^{1}H NMR spectra, we estimated the cross-linking ratios between the SSQ unit and the bipyridine ligand from the rates of amide-bond formation because we were not able to discriminate the mono and bis-substituted bipyridine ligands. ^{1}H NMR ((CD_3)$_2$SO, 400 MHz): δ 9.18 (br, 2H), 8.44 (br, 4H), 8.16 (br, 2H), 3.30 (br, 2H), 2.81 (br, 2H), 1.71 (br, 2H), 0.74 (br, 2H). ^{29}Si NMR((CD_3)$_2$SO, 80 MHz): δ–66.5.

Preparation of polymer composites. The mixture (10 mL) containing 100 mg of SSQ network polymers and various amounts of lanthanoid chloride salts in deionized water was stirred at room temperature for 1 day. Then the 300 μL solution was dropped on the quartz plate (1 cm × 5 cm), and the sample was irradiated at 50W of microwave for 2h for removing solvents. After drying *in vacuo*, the resulting films on quartz plate were used for following measurements.

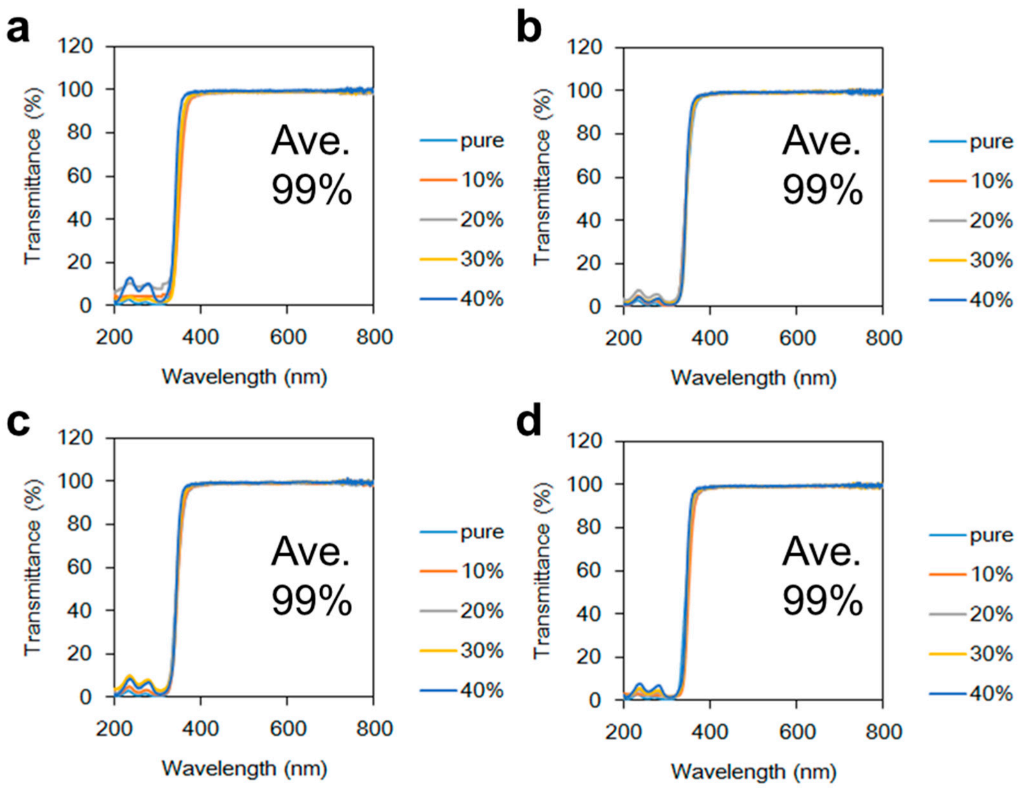

Figure S1. Transparency of the films containing 40 wt% (a) La(III), (b) Ce(III), (c) Gd(III) and (d) Yb(III) salts. Averaged values were calculated from 400 nm to 800 nm.

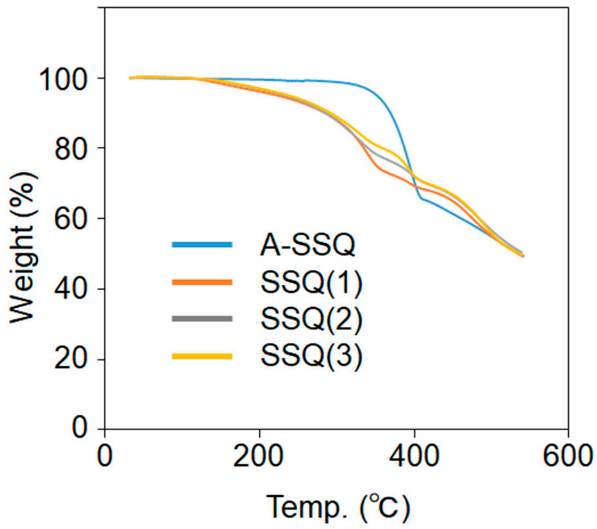

Figure S2. TGA profiles of SSQ networks.

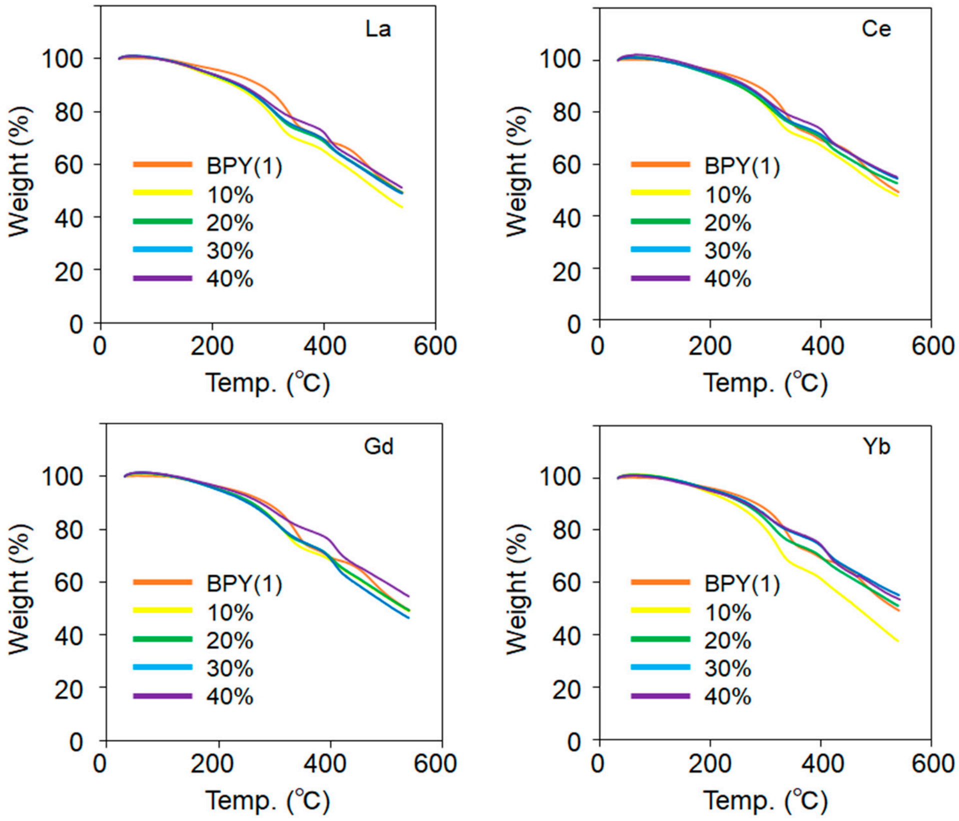

Figure S3. TGA profiles of SSQ networks BPY(2) with various concentrations of metal cations.

Table S1. Thermal stability of network polymers

SSQ	T_{d5} (°C)	T_{d20} (°C)
Amino	352	387
BPY(1)	224	335
BPY(2)	233	340
BPY(3)	238	360

Table S2. Thermal stability of hybrid films with variable cation concentrations

	La				Ce			
wt %	T_{d5} (°C)	ΔT_{d5} (°C)	T_{d20} (°C)	ΔT_{d20} (°C)	T_{d5} (°C)	ΔT_{d5} (°C)	T_{d20} (°C)	ΔT_{d20} (°C)
0	224		335		224			
10	179	–45	300	–35	195	–29	311	–24
20	188	–36	309	–26	193	–31	317	–18
30	188	–36	311	–24	200	–24	323	–12
40	188	–36	322	–13	212	–12	333	–2

	Gd				Yb			
wt %	T_{d5} (°C)	ΔT_{d5} (°C)	T_{d20} (°C)	ΔT_{d20} (°C)	T_{d5} (°C)	ΔT_{d5} (°C)	T_{d20} (°C)	ΔT_{d20} (°C)
0	224		335		224		335	
10	201	–23	314	–21	192	–32	302	–33
20	202	–22	314	–21	207	–17	318	–17
30	199	–25	315	–20	212	–12	340	+5
40	212	–12	356	+21	202	–22	344	+9
